# An easy synthesis of 5-functionally substituted ethyl 4-amino-1-aryl- pyrazolo-3-carboxylates: interesting precursors to sildenafil analogues

**DOI:** 10.1186/1860-5397-3-15

**Published:** 2007-05-01

**Authors:** Said A S Ghozlan, Khadija O Badahdah, Ismail A Abdelhamid

**Affiliations:** 1 Department of Chemistry, Faculty of Science, Cairo University, Giza, A. R. Egypt; 2 Department of Chemistry, Faculty of Science, King AbdulAziz University, Jeddah-21411. P.O. Box 154, Saudi Arabia

## Abstract

3-Oxo-2-arylhydrazononitriles **1a-c** react readily with chloroacetonitrile, ethyl chloroacetate, and with phenacyl chloride to give 4-aminopyrazoles **4a-e**. The pyrazolo[4,3-*d*]pyrimidine derivatives **7** and **10** are synthesized via reaction of the aminopyrazole **4b** with phenylisothiocyanate and DMFDMA/NH_4_OAc respectively.

## Background

**Scheme 1:**
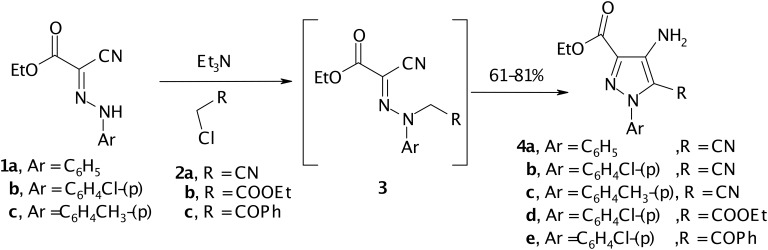
synthesis of Ethyl 4-amino-5-substituted-1-aryl-1*H*-pyrazole-3-carboxylates (**4**)

Interest in the chemistry of 4-aminopyrazole carboxylic acid derivatives has recently been recognized as their derivatives are ideal precursors for the synthesis of biologically active pyrazolo[4,3-*d*]pyrimidine ring systems [1–6]. The reported synthetic approaches to these derivatives are also multistep, non atom economical and non eco friendly [1,5–6]. Recently however a route to 4-aminopyrazole-5-carboxylic acid derivatives via reacting 2-arylhydrazononitriles with α-haloacid derivatives has been reported by Elnagdi et al [7–8] as well as other researchers [9]. In the present article we report results of our work aimed at exploring this synthetic methodology and adoption of products for the synthesis of pyrazolo[4.3-*d*]pyrimidines. Thus, compounds **1a-c**, were prepared according to literature procedures via coupling of ethyl cyanoacetate with aromatic diazonium salts [10]. It has been found that **1a-c** react with α-chloroacetonitrile **2a** to yield **4a-c**, most likely via acyclic intermediates **3a-c** that could not be isolated. The structure of **4a-c** was confirmed based on ^1^H NMR spectra that revealed the presence of amino signals and also ^13^C NMR which revealed the presence of only one CN signal. Similarly reacting **1b** with ethyl chloroacetate **2b** and with phenacyl chloride **2c** afforded **4d,e**. The structure of **4d,e** was also confirmed based on IR and ^13^C NMR, which revealed the absence of CN bands and signals (cf. Scheme 1).

**Scheme 2:**
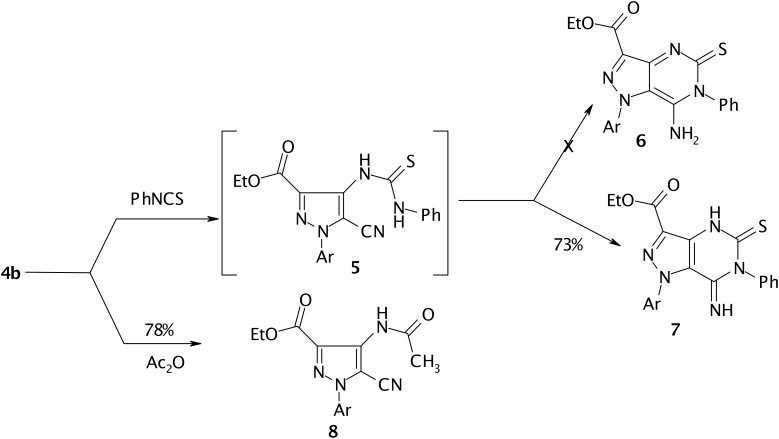
Reactivity of pyrazole **4b** with phenylisothiocyanate and acetic anhydride

Compound **4b** reacted readily with phenylisothiocyanate to yield a 1:1 adduct. The IR and ^13^C NMR spectra of the product revealed the absence of CN bands and signals. Thus structure **6** or **7** is suggested. ^1^H NMR showed two NH signals at δ 8.33 and 10.3 ppm, thus structure **7** is assigned for the reaction product. Acetylation of **4b** in acetic anhydride afforded monoacetyl derivative **8**. (cf. Scheme 2)

**Scheme 3:**
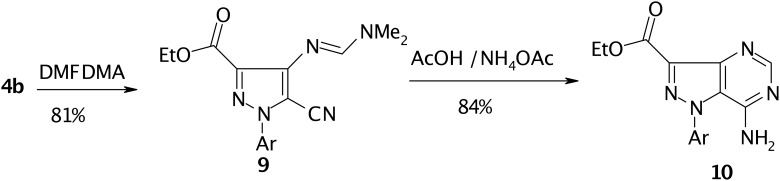
Conversion of pyrazole **4b** Ethyl into pyrazolo[4,3-*d*]pyrimidine-3-carboxylate **10**

Compound **4b** condensed with dimethylformamide dimethylacetal (DMFDMA) to yield the enamine **9**. The ^1^H NMR spectrum indicated two distinct singlets at ä 2.97 and 3.05 ppm for the *N*,*N*-dimethylamino protons which mean that the two methyl groups are magnetically nonequivalent, as to be expected. Compound **9** could be readily converted into pyrazolo[4,3-*d*]pyrimidine **10** on treatment with AcOH/NH_4_OAc mixture. (cf. Scheme 3)

**Scheme 4:**
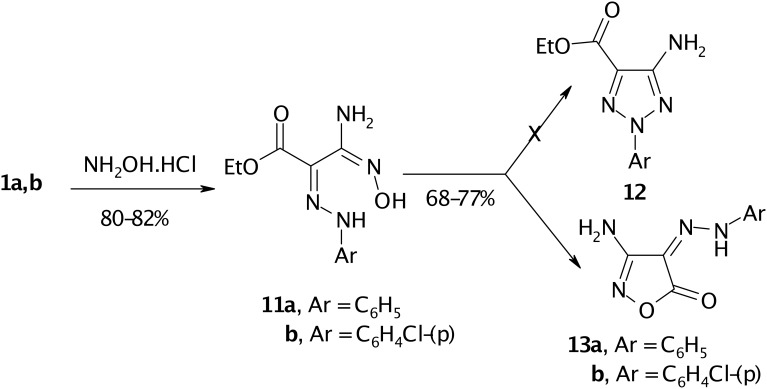
Conversion of arylhydrazononitriles **1** into 3-Amino-4-arylhydrazono-4*H*-isoxazol-5-one

Compound **1** reacted with hydroxylamine hydrochloride in ethanol/sodium acetate solution to yield amidooxime **11** as in the literature [10]. Trials to cyclize the amidooxime into 1,2,3-triazole **12** utilizing the reaction conditions described earlier in literature [11] failed. However, the amidooxime **11** cyclizes smoothly via loss of ethanol in DMF and in presence of anhydrous sodium acetate into isoxazolone **13**. (cf. Scheme 4)

## Conclusion

We could show that arylhydrazononitriles **1a-c** are valuable precursors to 4-amino-5-substituted-1-aryl-1H-pyrazole-3-carboxylic acid ethyl ester which can be used for preparation of sildenafil analogues.

## Supporting Information

File 1 The experimental section. The experimental data and the results of analysis
