# Supplementary material for: An easy synthesis of 5-functionally substituted ethyl 4-amino-1-aryl- pyrazolo-3-carboxylates: interesting precursors to sildenafil analogues
Source: Beilstein J Org Chem. 2007 May 1;3:15. doi: 10.1186/1860-5397-3-15 (PMC1885264; doi:10.1186/1860-5397-3-15)
Supplement: File 1 — The experimental section. The experimental data and the results of analysis [file Beilstein_J_Org_Chem-03-15-s001.doc]

**Experimental:**

### All melting points were determined on a Stuart melting point apparatus and are uncorrected. The ^1^H and ^13^C NMR spectra were recorded at (300 MHz) and (75 MHz) respectively were recorded on Varian Gemini NMR spectrometer. Chemical shifts (δ) are given from TMS (0 ppm) as internal standard for ^1^H-NMR and ^13^C-NMR. Mass spectra were measured on a Shimadzu GMMS -QP-1000 EX mass spectrometer at 70 eV. The elemental analyses were performed at the micro analytical center, Cairo University. The IR spectra were recorded in KBr using a FTIR unit Bruker-vector 22 spectrophotometer.

**General method for preparation of Ethyl 4-amino-5-substituted-1-aryl-1*H*-pyrazole-3-carboxylates (4)**

To a solution of **1a-c** (0.1 mol) and triethylamine (20 ml) chloroacetonitriles, ethyl chloroacetate, or phenacyl chloride (0.2 mol) was added. The reaction mixture was the refluxed for 45 minute, and then poured onto ice-cold water and HCl, the solid product so formed was filtered off and crystallized from ethanol to afford **4**.

**Ethyl 4-mino-5-cyano-1-phenyl-1*H*-pyrazole-3-carboxylate (4a)**

Yield 2 g (78 %); m.p: 140-142 ^o^C, ^1^H MNR (300 MHz, DMSO-d6): δ, ppm: 1.31 (t, 3H, CH_3_, *J* = 7.2 Hz), 4.35 (q, 2H, CH_2_, *J* = 7.2 Hz), 6.14 (s, 2H, NH_2_), 7.52-7.70 (m, 5H, Ar-H); ^13^C NMR (DMSO-d6): δ, ppm: 14.14, 60.51, 98.83, 111.11, 122.78, 129.12, 129.58, 129.71, 138.02, 144.18, 161.75 ;MS (EI): m/z (%) = 256 (M^+^); IR (KBr, cm^-1^): υ_max_ = 3444 and 3349 (NH_2_), 2219 (CN), 1696 (CO); *Anal*. Calcd. for C_13_H_12_N_4_O_2_ (256.27): C, 60.93; H, 4.72; N, 21.86. Found: C, 61.27; H, 4.57; N, 21.76.

**Ethyl 4-amino-1-(4-chlorophenyl) -5-cyano-1*H*-pyrazole-3-carboxylate (4b)**

Yield 2.35 g (81 %); m.p: 173-175 ^o^C; ^1^H MNR (300 MHz, DMSO-d6): δ, ppm: 1.31 (t, 3H, CH_3_, *J* = 7.2 Hz), 4.33 (q, 2H, CH_2_, *J* = 7.2 Hz), 6.17 (s, 2H, NH_2_), 7.67 (d, 2H, Ar-H, *J* = 8.4 Hz), 7.72 (d, 2H, Ar-H, *J* = 8.4 Hz); ^13^C NMR (DMSO-d6): δ, ppm: 14.1, 60.6, 98.7, 111, 124.4, 129.6, 13, 133.5, 136.8, 144.2, 161.6; MS (EI): m/z (%) = 290 (M^+^);

IR (KBr, cm^-1^): υ_max_ = 3488 and 3384 (NH_2_), 2217 (CN), 1700 (CO); *Anal*. Calcd. for C_13_H_11_ClN_4_O_2_ (290.71): C, 53.71; H, 3.81; N, 19.27. Found: C, 53.97; H, 4.04; N, 19.11.

**Ethyl 4-amino-5-cyano-1-*p*-tolyl- 1*H*-pyrazole-3-carboxylate (4c)**

Yield 2 g (75 %); m.p: 168-170 ^o^C; ^1^H MNR (300 MHz, DMSO-d6): δ, ppm: 1.31 (t, 3H, CH_3_, *J* = 7.2 Hz), 4.32 (q, 2H, CH_2_, *J* = 7.2 Hz), 6.10 (s, 2H, NH_2_), 7.38 (d, 2H, Ar-H, *J* = 8.4 Hz),7.56 (d, 2H, Ar-H, *J* = 8.4 Hz); ^13^C NMR (DMSO-d6): δ, ppm: 14.1, 20.5, 60.4, 98.9, 111.1, 122.6, 129.4, 129.9, 135.7, 138.9, 144.1, 161.8 ;MS (EI): m/z (%) = 270 (M^+^); IR (KBr, cm^-1^): υ_max_ = 3440 and 3345 (NH_2_), 2218 (CN), 1693 (CO); *Anal*. Calcd. for C_14_H_14_N_4_O_2_ (270.29): C, 62.21; H, 5.22; N, 20.73. Found: C, 62.52; H, 5.09; N, 20.54.

**Diethyl 4-amino-1-(4’-chlorophenyl)-1*H*-pyrazole-3,5-dicarboxylate (4d)**

Yield 2.06 g (61 %); m.p: 138-140 ^o^C; ^1^H MNR (300 MHz, DMSO-d6): δ, ppm: 1.13 (t, 3H, CH_3_, *J* = 7.2 Hz), 1.30 (t, 3H, CH_3_, *J* = 7.2 Hz), 4.16 (q, 2H, CH_2_, *J* = 7.2 Hz), 4.31 (q, 2H, CH_2_, *J* = 7.2 Hz), 5.69 (s, 2H, NH_2_), 7.48-7.56 (m, 4H, Ar-H); ^13^C NMR (DMSO-d6): δ, ppm: 13.78, 14.6, 60.3, 60.3, 116.3, 127.6, 128.3, 129.0, 133.0, 139.0, 141.2, 158.6, 162.2; MS (EI): m/z (%) = 337 (M^+^); IR (KBr, cm^-1^): υ_max_ = 3490 and 3383 (NH_2_), 1709, 1604 (2 CO); *Anal*. Calcd. for C_15_H_16_ClN_3_O_4_ (337.77): C, 53.34; H, 4.77; N, 12.44. Found: C, 53.11; H, 4.92; N, 12.14.

**Ethyl 4-amino-5-benzoyl-1-(4’-chlorophenyl)-1*H*-pyrazole-3-carboxylate (4e)**

Yield 2.47 g (67 %); m.p: 138-140 ^o^C; ^1^H MNR (300 MHz, DMSO-d6): δ, ppm: 1.32 (t, 3H, CH_3_, *J* = 7.2 Hz), 4.38 (q, 2H, CH_2_, *J* = 7.2 Hz), 5.85 (s, 2H, NH_2_), 7.23-7.47 (m, 9H, Ar-H); ^13^C NMR (DMSO-d6): δ, ppm: 14.2, 60.4, 124.7, 126.3, 128.1, 128.4, 128.52, 129.8, 132, 132.2, 137.5, 138.6, 141.7, 162.2, 185.1 ;MS (EI): m/z (%) = 369 (M^+^); IR (KBr, cm^-1^): υ_max_ = 3478 and 3369 (NH_2_), 1702 (CO); *Anal*. Calcd. for C_19_H_16_ClN_3_O_3_ (369.81): C, 61.71; H, 4.36; N, 11.36. Found: C, 62.02; H, 4.22; N, 11.37..

**Ethyl7-imino-6-phenyl-1-(4’-chlorophenyl)-5-thioxo-4,5,6,7-tetrahydro-1*H*-pyrazolo[4,3-*d*]pyrimidine-3-carboxylylate (7)**

To a solution of **4b** (0.01 mol) and (20 ml) of pyridine, phenylisothiocyanate (0.1mol) was added. The reaction mixture was then refluxed for 5 hours then cooled to room temperature and poured onto cold water and hydrochloric acid mixture, the solid product so formed was filtered off and crystallized from ethanol to afford **7**.

Yield 3.1 g (73 %); m.p  ^o^C; ^1^H MNR (300 MHz, DMSO-d6): δ, ppm: 1.35 (t, 3H, CH_3_, *J* = 7.2 Hz), 4.39 (q, 2H, CH_2_, *J* = 7.2 Hz), 6.87-7.77 (m, 9H, Ar-H), 8.33 (br s, 1H, NH), 10.3 (br s, 1H, NH); ^13^C NMR (DMSO-d6): δ, ppm: 14.1, 61.3, 120.6, 121.4, 123, 123.5, 123.8, 126.7, 128.2, 128.5, 133.7, 135.3, 137.5, 146.9, 159.9, 163.1; MS (EI): m/z (%) = 425 (M^+^); IR (KBr, cm^-1^): υ_max_ = 3387, 3206 (2 NH), 1744 (CO), 1699 (CS); *Anal*. Calcd. for C_20_H_16_ClN_5_O_2_S (425.90): C, 56.40; H, 3.79; N, 16.44. Found: C, 56.67; H, 3.93; N, 16.34.

**Ethyl 4-acetylamino--5-cyano-1-(4’-chlorophenyl)-1*H*-pyrazole-3-carboxylate (8)**

### 10 mmol of pyrazole 4b was refluxed in acetic anhydride (20 ml) for 2 h. The solvent was evaporated under vacuum and the crude product was collected and crystallized from ethanol.

Yield 2.6 g (78 %); m.p: 118-120 ^o^C; ^1^H MNR (300 MHz, DMSO-d6): δ, ppm: 1.28 (t, 3H, CH_3_, *J* = 7.2 Hz), 2.16 (s, 3H, CH_3_), 4.33 (q, 2H, CH_2_, *J* = 7.2 Hz), 7.68 (d, 2H, Ar-H, *J* = 8 Hz), 7.94(d, 2H, Ar-H, *J* = 8 Hz) 10.07 (s, 1H, NH); ^13^C NMR (DMSO-d6): δ, ppm: 13.9, 25.8, 61.6, 110.4, 116, 125.7, 129.7, 130.6, 134.5, 135.9, 139.9, 160.2, 171.5; MS (EI): m/z (%) = 332 (M^+^); IR (KBr, cm^-1^): υ_max_ = 3332 (NH), 2235 (CN), 1725 (COOEt, 1691 (NHCO); *Anal*. Calcd. for C_15_H_13_ClN_4_O_3_ (332.75): C, 54.15; H, 3.94; N, 16.84. Found: C, 54.35; H, 4.02; N, 16.68.

**Ethyl 4-(dimethylamino-methyleneamino)-5-cyano-1-(4’-chlorophenyl))-1*H*-pyrazole-3-carboxylate (9)**

A mixture of compound **4b** (0.01 mol), and dimethylformamide dimethylacetal (0.01 mol) in dioxan (20 ml) was heated under reflux for 5 hours, then left to cool and poured into water. The solid product obtained was crystallized from ethanol.

Yield 2.8 g (81 %); m.p: 120-122  ^o^C; ^1^H MNR (300 MHz, DMSO-d6): δ, ppm: 1.25 (t, 3H, CH_3_, *J* = 7.2 Hz), 2.97 (s, 3H, CH_3_), 3.05 (s, 3H, CH_3_), 4.28 (q, 2H, CH_3_, *J* = 7.2 Hz), 7.67-7.77 (m, 4H, Ar-H), 7.97 (s, 1H, amidine-H); ^13^C NMR (DMSO-d6): δ, ppm: 13.9, 33.6, 60.5, 107.3, 111.1, 124.4, 129.7, 133.6, 135.1, 136.8, 147.6, 157.7, 160.7 ;MS (EI): m/z (%) = 345 (M^+^); IR (KBr, cm^-1^): υ_max_ = 2223 (CN), 1722 (COOEt; *Anal*. Calcd. for C_16_H_16_ClN_5_O_2_ (345.79): C, 55.58; H, 4.66; N, 20.25. Found: C, 55.63; H, 4.81; N, 20.46.

**Ethyl 7-amino-1-(4’-chlorophenyl)-1*H*-pyrazolo[4,3-*d*]pyrimidine-3-carboxylate (10)**

A mixture of compound **9** (0.01 mol), and ammonium acetate (2g) in acetic acid (20 ml) was heated under reflux for 3 hours, then left to cool and poured onto water. The solid product obtained was crystallized from ethanol.

Yield 2.67 g (84 %); m.p: 190-191 ^o^C; ^1^H MNR (300 MHz, DMSO-d6): δ, ppm: 1.31 (t, 3H, CH_3_, *J* = 7.2 Hz), 4.35 (q, 2H, CH_2_, *J* = 7.2 Hz), 6.80 (s, 2H, NH_2_), 7.63-7.74 (m, 4H, Ar-H), 8.40 (s, 1H, pyrimidine-H); ^13^C NMR (DMSO-d6): δ, ppm: 14.2, 60.6, 121.4, 124.4, 127.4, 129.6, 134.1, 137.4, 143.2, 144.2, 153.9, 160.5; MS (EI): m/z (%) = 317 (M^+^); IR (KBr, cm^-1^): υ_max_ = 3396 and 3321 (NH_2_), 1715 (CO); *Anal*. Calcd. for C_14_H_12_ClN_5_O_2_ (317.74): C, 52.92; H, 3.81; N, 22.04. Found: C, 53.13; H, 3.73; N, 22.23..

**General method for preparation of (N-Hydroxycarbamidoyl)-(4’chlorophenylhydrazono)- acetate (11)**

### A mixture of arylhydrazononitriles 1 (10 mmol), and hydroxyl amine hydrochloride was refluxed in ethanol (20 ml) in presence of sodiumacetate for 2 h. The solvent was evaporated under vacuum and the crude product was collected and crystallized from ethanol /Dioxan.

**(*N*-Hydroxycarbamimidoyl)-(phenylhydrazono)acetic acid ethyl ester (11a)**

Yield 2.0 g (80 %); m.p: 208-210  ^o^C; ^1^H MNR (300 MHz, DMSO-d6): δ, ppm: 1.29 (t, 3H, CH_3_, *J* = 7.2 Hz) 4.25 (q, 2H, CH_2_, *J* = 7.2 Hz), 6.43 (s, 2H, NH_2_), 7.04-7.72 (m, 4H, Ar-H), 10.23 (s, 1H, OH), 13.36 (s, 1H, NH); MS (EI): m/z (%) = 250 (M^+^); IR (KBr, cm^-1^): υ_max_ = 3465 and 3358 (NH_2_), 3032 (br OH); *Anal*. Calcd. for C_11_H_14_N_4_O_3_ (250.26): C, 52.79; H, 5.64; N, 22.39. Found: C, 52.23; H, 5.44; N, 22.45.

**[(4-Chlorophenyl)hydrazono)](*N*-Hydroxycarbamimidoyl)-acetic acid ethyl ester (11b)**

Yield 2.33 g (82 %); m.p: 212-213 ^o^C; ^1^H MNR (300 MHz, DMSO-d6): δ, ppm: 1.28 (t, 3H, CH_3_, *J* = 7.2 Hz) 4.32 (q, 2H, CH_2_, *J* = 7.2 Hz), 6.58 (s, 2H, NH_2_), 6.98-7.96 (m, 4H, Ar-H), 12.4 (s, 1H, OH), 13.46 (s, 1H, NH); MS (EI): m/z (%) = 284 (M^+^); IR (KBr, cm^-1^): υ_max_ = 3473 and 3360 (NH_2_), 3036 (br OH), 1693 (CO); *Anal*. Calcd. for C_11_H_13_ClN_4_O_3_ (284.70): C, 46.41; H, 4.60; N, 19.68. Found: C, 46.52; H, 4.46; N, 19.73.

**General method for preparation of 3-Amino-4-arylhydrazono-4*H*-isoxazol-5-ones (13)**

### A mixture of arylhydrazononitriles 1 (10 mmol), and hydroxyl amine hydrochloride was refluxed in DMF (20 ml) in presence of anhydrous sodiumacetate (2g) for 8 h. The solvent was evaporated under vacuum and the crude product was collected and crystallized from acetic acid.

**3-Amino-4-(phenylhydrazono)-4*H*-isoxazol-5-one (13a)**

Yield 1.57 g (77 %); m.p: 192-194 ^o^C; ^1^H MNR (300 MHz, DMSO-d6): δ, ppm: 6.43 (s, 2H, NH_2_), 7.06-7.72 (m, 5H, Ar-H), 12.44 (s, 1H, NH); ^13^C NMR (DMSO-d6): δ, ppm: 115.20, 115.89, 116.30, 125.34, 129.22, 141.69, 158.95, 162.34; MS (EI): m/z (%) = 204 (M^+^); IR (KBr, cm^-1^): υ_max_ = 3432 (NH), 3335 and 3265 (NH_2_), 1685 (CO); *Anal*. Calcd. for C_9_H_8_N_4_O_2_ (204.19): C, 52.94; H, 3.95; N, 27.44. Found: C, 52.78; H, 3.73; N, 27.72.

**3-Amino-4-[(4’-chlorophenyl)hydrazono]-4*H*-isoxazol-5-one (13b)**

Yield 1.62 g (68 %); m.p: 240-242 ^o^C; ^1^H MNR (300 MHz, DMSO-d6): δ, ppm: 7.38-8.26 (m, 4H, Ar-H), 23.48 (s, 2H, NH_2_), 15.81 (s, 1H, NH); MS (EI): m/z (%) = 238 (M^+^); IR (KBr, cm^-1^): υ_max_ = 3478 (NH), 3346 and 3258 (NH_2_), 1697 (CO); *Anal*. Calcd. for C_9_H_7_ClN_4_O_2_ (238.63): C, 45.30; H, 2.96; N, 23.48. Found: C, 45.47; H, 3.12; N, 23.32.
